# Patients in intensive care unit for COVID-19 pneumonia: the lung ultrasound patterns at admission and discharge. An observational pilot study

**DOI:** 10.1186/s13089-021-00213-x

**Published:** 2021-02-24

**Authors:** Paolo Persona, Ilaria Valeri, Francesco Zarantonello, Edoardo Forin, Nicolò Sella, Giulio Andreatta, Christelle Correale, Eugenio Serra, Annalisa Boscolo, Giovanni Volpicelli, Paolo Navalesi

**Affiliations:** 1 Institute of Anesthesia and Critical Care, Padua University Hospital, Via V. Gallucci, 13, 35121 Padova, Italy; 2 Anesthesia and Critical Care, Department of Medicine-DIMED, University of Padua, Padua, Italy; 3 Department of Emergency Medicine, San Luigi Gonzaga University Hospital, Torino, Italy

## Abstract

**Background:**

During COVID-19 pandemic, optimization of the diagnostic resources is essential. Lung Ultrasound (LUS) is a rapid, easy-to-perform, low cost tool which allows bedside investigation of patients with COVID-19 pneumonia. We aimed to investigate the typical ultrasound patterns of COVID-19 pneumonia and their evolution at different stages of the disease.

**Methods:**

We performed LUS in twenty-eight consecutive COVID-19 patients at both admission to and discharge from one of the Padua University Hospital Intensive Care Units (ICU). LUS was performed using a low frequency probe on six different areas per each hemithorax. A specific pattern for each area was assigned, depending on the prevalence of A-lines (A), non-coalescent B-lines (B1), coalescent B-lines (B2), consolidations (C). A LUS score (LUSS) was calculated after assigning to each area a defined pattern.

**Results:**

Out of 28 patients, 18 survived, were stabilized and then referred to other units. The prevalence of C pattern was 58.9% on admission and 61.3% at discharge. Type B2 (19.3%) and B1 (6.5%) patterns were found in 25.8% of the videos recorded on admission and 27.1% (17.3% B2; 9.8% B1) on discharge. The A pattern was prevalent in the anterosuperior regions and was present in 15.2% of videos on admission and 11.6% at discharge. The median LUSS on admission was 27.5 [21–32.25], while on discharge was 31 [17.5–32.75] and 30.5 [27–32.75] in respectively survived and non-survived patients. On admission the median LUSS was equally distributed on the right hemithorax (13; 10.75–16) and the left hemithorax (15; 10.75–17).

**Conclusions:**

LUS collected in COVID-19 patients with acute respiratory failure at ICU admission and discharge appears to be characterized by predominantly lateral and posterior non-translobar C pattern and B2 pattern. The calculated LUSS remained elevated at discharge without significant difference from admission in both groups of survived and non-survived patients.

## Background

Intensive care unit (ICU) admission is required in up to 16% of positive tested patients for novel SARS-CoV-2 [1]. In the current COVID-19 outbreak, this percentage overwhelms the health care system capabilities and thus requires a smart resource optimization. Chest imaging plays a crucial role in the diagnosis and in the management of patients with COVID-19 pneumonia. CT scan is considered the gold standard imaging modality for the investigation of patients with COVID-19 interstitial pneumonia [2], but its routine applicability is limited especially in critically ill patients and by consideration of its cost-effectiveness balance [3], particularly because of the need to transfer unstable patients to the radiology department, the high costs and, of no minor concern, because of the risk of personal and environmental viral spread.

**Fig. 1:**
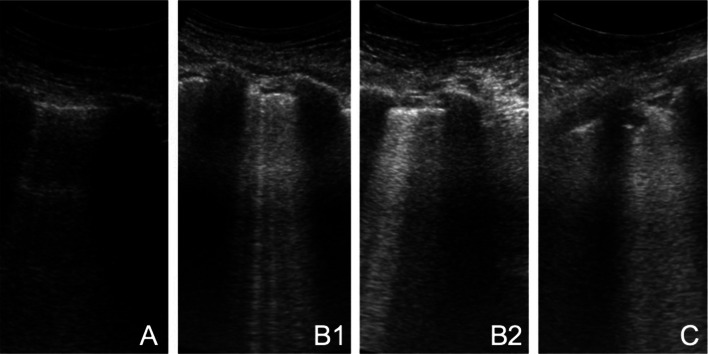
Common Lung Ultrasound patterns in COVID-19 patients. From left to right: (** a, b1, b2, c**) pattern

Lung ultrasonography (LUS) is a rapid, bedside tool that has demonstrated to be more accurate than chest radiograph in the identification of the main pulmonary lesions of critically ill patients affected by acute respiratory distress syndrome (ARDS) [4]. LUS probed to be useful to measure the degree of lung aeration [5] (Fig. 1). In particular, the LUS score (LUSS) has been validated for monitoring lung aeration in ARDS patients with a good correlation with chest CT scan [6] and for prediction of post extubation distress [7].

Peng and colleagues were the first to describe general LUS findings in twenty patients affected by COVID-19 pneumonia, but they did not define neither the timing of LUS acquisition (at admission, during the hospital stay or discharge) nor the setting (emergency department, medical ward, ICU) [8]. LUS signs and patterns of COVID-19 pneumonia have been described during the peak of the pandemic in Italy and Spain [9–11]. A recent study detailed the LUS findings in critically ill patients [12].

The aim of the present study was to describe the LUS patterns and their evolution at different timing from ICU admittance to discharge in a series of critically ill patients with confirmed COVID-19 pneumonia and severe hypoxemic acute respiratory failure (hARF).

We observed the initial LUS patterns, analysed quantification and distribution of the pulmonary damage in different lung regions and compared its evolution over time from admission to discharge from the ICU.

## Methods

We prospectively investigated all consecutive COVID-19 patients with hARF admitted to Padua University Hospital ICU between March and April 2020 (Table 1). Local ethical board approval was obtained (no 0021712). Exclusion criteria were age < 18 years old, history of lung resections or pneumonectomy, neoplastic lung disease or metastatic lung localizations and severe pulmonary fibrosis. LUS was performed at admission in ICU and on the day of discharge. The discharge LUS examination in survived patients, who had been successfully extubated and/or did not need non-invasive ventilation anymore, was performed before transfer from ICU to less intensive care departments, either sub intensive care unit or general ward. For the patients who died in ICU, we considered the last collected videos before death, as we performed and recorded LUS once daily in each patient in supine position. For each patient, six videos of 5 s per hemithorax were collected (anterosuperior, anteroinferior, laterosuperior, lateroinferior, posterosuperior and posteroinferior zones) using a low frequency convex probe (1.4–5.1 MHz, C5 1-S, Mindray M9; depth of 15 cm and focus on pleural line) and without activation of tissue harmonic imaging. LUS was performed adopting the longitudinal scanning technique, the probe was therefore set perpendicularly to the ribs and tilted to get the optimal view10. Acquisition of posterior images was obtained rotating the patient 45 degrees to the contralateral side for each side. Considering that in each of the 6 areas per side may be studied 2–3 intercostal spaces, the assigned pattern was the worst observed one. Recorded clips were anonymized and each patient was identified by an individual code, which was stored in a safe place by one investigator. The qualitative analysis was blindly performed offline by two reviewers (PP e IV) expert in LUS. If in doubt, a third examiner was involved (FZ). Interobserver variability was assessed by Cohen's Kappa.

**Table 1:** General characteristics of COVID-19 patients on admission and Lung Ultrasound (LUS) score at admission and discharge

Gender, male	21/28 (75%)	EF (%)	50 [45–55]
Age (years)	69 [61–77]	MAP (mmHg)	83 [73.3–86.3]
BMI (kg/m^2^)	27.5 [24.5–32.6]	HR (bpm)	80 [69.3–85.5]
Time from onset of symptoms to ICU admission (days)	9 [7–12.25]	Vasoactive drugs	10/28 (35.7%)
NIV on admission	16/28 [57.2%]	Age-adjusted Charlson Comorbidity Index	4 [2.75–4.25]
ETT on admission	12/28 [42.8%]	SOFA score	3 [2–5]
PaO_2_/FiO_2_ ratio	146.5 [111.5–152.5]	Pct ug/L (0–0.5)	0.34 [0.09–1.06]
PEEP (cmH2O)	12 [10–12]	proBNP ng/L (0–100)	55 [31–124]
Admission		Discharge	
Global LUS score	27.5 [21–32.25]	Global LUS score	31 [24.75–33]
LUS score survivors	28 [20.5–32]	LUS score survivors	31 [17.5–32.75]
LUS score non-survivors	25 [21–32.5]	LUS score non-survivors	30.5 [27–32.75]
LUS score right lung	13 [10.75–16]	LUS score right lung	14.5 [11.75–16.25]
LUS score left lung	15 [10.75–17]	LUS score left lung	16 [13–18]

Variables are expressed as median and interquartile range or percentage

*NIV* non-invasive ventilation, *ETT* endotracheal tube, *EF* ejection fraction, *Pct* procalcitonin, *pro-BNP* brain natriuretic peptide, *SOFA* sequential organ failure assessment, *MAP* mean arterial pressure, *HR* heart rate, *BMI* body mass index

The four considered patterns were already detailed in literature [10]: normal-A-lines; B1–non-coalescent B-lines; B2–coalescent B-lines and “light beam”; C–consolidation. LUSS was calculated as the sum of the score of each area (normal = 0, B1 = 1, B2 = 2, and *C* = 3, ranging from 0 to 36). Data are expressed as percentage or median and interquartile range. Mann–Whitney test was applied to compare median values.

## Results

**Fig. 2:**
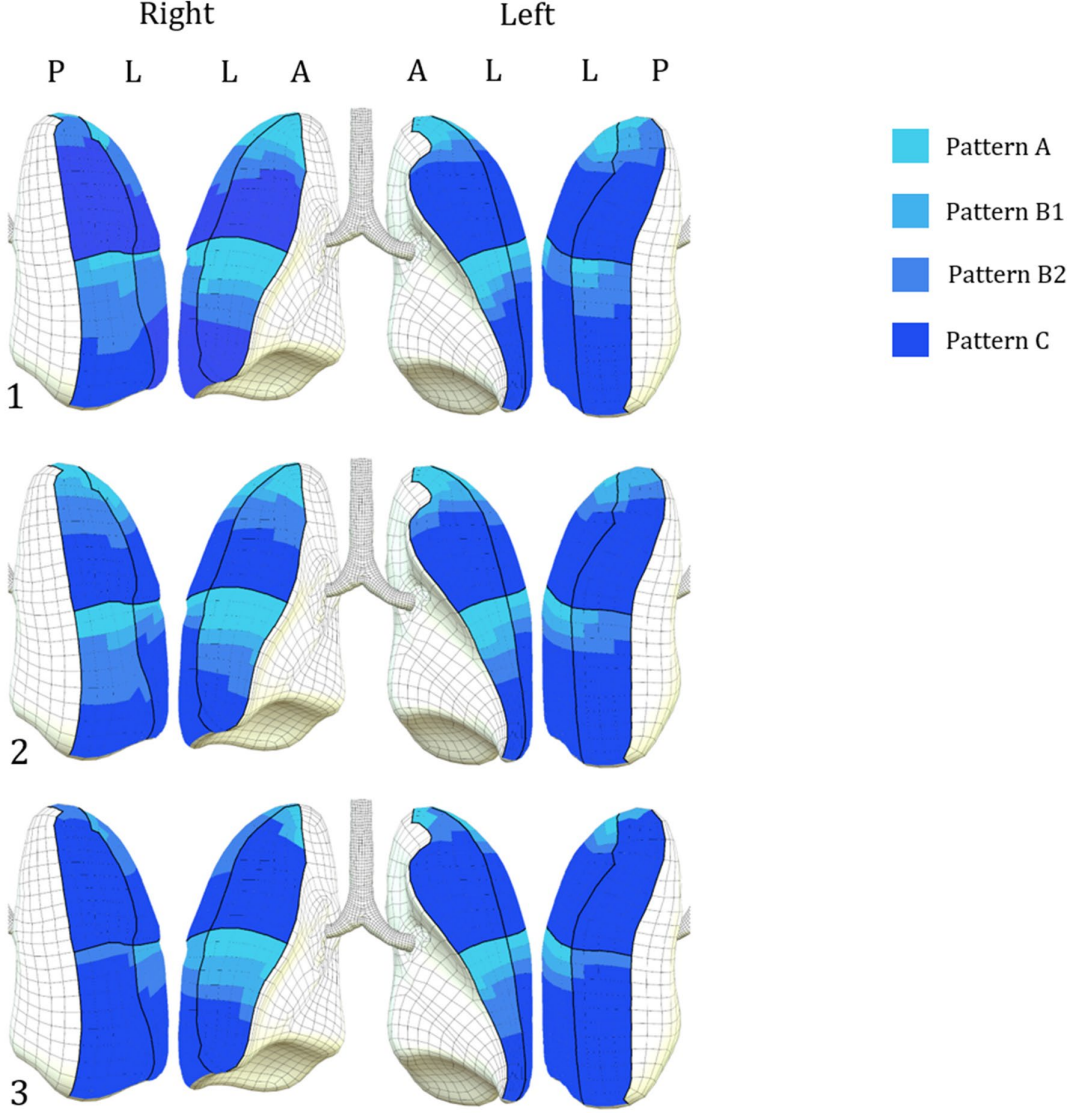
LUS patterns frequency distribution on the twelve investigated areas on the day of ICU admission (1) and at discharge considering survived (2) and non-survived patients (3): the number of squares of each colour is proportional to the percentage of presentation of each pattern in the considered region. Examined regions were posterior (**p**), lateral (**l**), split between the front and back views, and anterior (**a**) for both hemithoraces, each one divided into superior and inferior

Demographic data and general characteristics of patients on admission are shown in Table 1. Median length of ICU stay was 8 days [4.5–16.75]. Mortality was 35.7% (10 out of 28 patients). The agreement between the two reviewers showed a Cohen's Kappa > 0.9. Prevalence and distribution of LUS patterns on admission and discharge are shown in Table 2 and LUS patterns frequency distribution on the twelve investigated areas on admission, on discharge and on the day of death are graphically depicted in Fig. 2.

**Table 2:** Frequency of presentation of different lung ultrasound patterns on each investigated area on admission and discharge in COVID-19 patients

On admission n. (%)	A pattern	B1 pattern	B2 pattern	C pattern
ASR	7 (25)	3 (10.7)	5 (17.9)	13 (46.4)
AIR	9 (32.1)	2 (7.1)	5 (17.9)	12 (42.6)
LSR	6 (21.4)	0	9 (32.1)	13 (46.4)
LIR	3 (10.7)	1 (3.6)	9 (32.1)	15 (53.6)
PSR	1 (3.6)	0	5 (17.9)	22 (78.6)
PIR	2 (7.1)	4 (14.3)	8 (28.6)	14 (50)
ASL	6 (21.4)	2 (7.1)	4 (14.2)	16 (57.1)
AIL	6 (21.4)	2 (7.1)	3 (10.7)	17 (60.7)
LSL	5 (17.9)	2 (7.1)	3 (10.7)	18 (64.2)
LIL	2 (7.1)	2 (7.1)	4 (14.2)	20 (71.4)
PSL	3 (10.7)	2 (7.1)	4 (14.2)	19 (67.8)
PIL	1 (3.6)	2 (7.1)	6 (21.4)	19 (67.8)
TOTAL n. (%)	51 (15.2)	22 (6.5)	65 (19.3)	198 (58.9)

On discharge *n*. (%)	A pattern	B1 pattern	B2 pattern	C pattern
ASR	5 (17.9)	2 (7.1)	6 (21.4)	15 (53.6)
AIR	7 (25)	4 (14.3)	5 (17.9)	12 (42.8)
LSR	4 (14.3)	3 (10.7)	5 (17.9)	16 (57.1)
LIR	1 (3.6)	4 (14.3)	6 (21.4)	17 (60.7)
PSR	2 (7.1)	2 (7.1)	6 (21.4)	18 (64.3)
PIR	3 (10.7)	1 (3.6)	7 (25)	17 (60.7)
ASL	5 (17.9)	2 (7.1)	4 (14.3)	17 (60.7)
AIL	6 (21.4)	3 (10.7)	6 (21.4)	13 (46.4)
LSL	3 (10.7)	3 (10.7)	4 (14.3)	18 (64.3)
LIL	3 (10.7)	3 (10.7)	3 (10.7)	19 (67.9)
PSL	0	5 (17.9)	2 (7.1)	21 (75)
PIL	0	1 (3.6)	4 (14.3)	23 (82.1)
TOTAL *n*. (%)	39 (11.6)	33 (9.8)	58 (17.3)	206 (61.3)

*ASR* anterosuperior right zone, *AIR* anteroinferior right zone, *LSR* laterosuperior right zone, *LIR* lateroinferior right zone, *PSR* posterosuperior right zone, *PIR* posteroinferior right zone, *ASL* anterosuperior left zone, *AIL* anteroinferior left zone, *LSL* laterosuperior left zone, *LIL* lateroinferior left zone, *PSL* posterosuperior left zone, *PIL* posteroinferior left zone

The median LUSS at admission was 27.5 [21–32.25], 28 [20.5–32] in survived and 25 [21–32.5] in non-survived patients (*p* = 1); at discharge the median LUSS was 31 [17.5–32.75] in survived patients and 30.5 [27–32.75] in non-survived group (*p* = 0.58). There were no differences between median LUSS at admission and discharge in survived (*p* = 0.88) and non-survived patients (*p* = 0.27). At admission the right hemithorax had a median LUSS of 13 [10.75–16] and the left hemithorax a median LUSS of 15 [10.75–17] (*p* = 0.19); at discharge the right hemithorax had a median LUSS of 14.5 [11.75–16.25] and the left one of 16 [13–18].

## Discussion

The main finding of our study is that LUSS at discharge in survived patients was still elevated and not significantly different from the score observed on the day of admission to ICU. The most represented LUS pattern in the total collected videos on admission was type C. All 28 patients showed non-translobar consolidations, mostly characterized by a mantellary distribution in posterior and lateral zones. These peripheral consolidations may be considered the hallmark of the COVID-19 pneumonia, together with the other signs of the interstitial acute damage. Similar consolidations are observed also in lung infarcts due to pulmonary embolism (PE), early stages of bacterial pneumonia, small subpleural lung abscess, metastasis and subpleural foci of lung cancer [13]. Thus, the differential diagnosis of the origin of these consolidations remains to be explored as it has been demonstrated that COVID-19 may be complicated by PE and bacterial cross infections [14].

However, in our cohort, procalcitonin values suggested a low probability of concomitant bacterial superinfection at ICU admission [15]. We believe LUS cannot differentiate if those consolidations are part of a patchy and multiform ongoing interstitial viral infection or if they are due to infarction in segmental or subsegmental embolism. Therefore, multiorgan integration of LUS with venous and cardiac ultrasound may be of help [16].

Peng et al. [8] reported a variety of patterns, including multifocal small, non-translobar, and translobar consolidations with occasional mobile air bronchograms. The same authors did not detail the prevalence of translobar consolidations with air bronchograms. We speculate that the prevalence of this LUS pattern could be influenced by the timing of LUS acquisition during the time course of the COVID-19 pneumonia, by its clinical severity and probability of superinfections. In our study only one patient, with a previous history of chronic renal failure, showed both peripheral consolidations and bilateral basal translobar consolidations with air bronchograms and bilateral large pleural effusion with elevated values of pro-BNP and procalcitonin on admission.

On the day of admission, the A pattern associated with normally aerated lung was mostly present in the anterior and superolateral lung regions. The more represented B pattern was the B2, in most cases observed by moving the probe from the anterior to the lateral and posterior areas in a quarter of the videos recorded at admission. The B-lines patterns were often associated with small subpleural subcentimetric echo-poor areas, had a dishomogeneous patchy distribution and were evident despite positive end-expiratory pressure (PEEP), which was given at a median value of 12 [10–12] cmH_2_O. These findings suggest a diffuse peripheral parenchymal lung damage that is not responsive to PEEP during the initial course of the disease in severe hARF and is unrelated to cardiogenic alveolar edema [17].

Similarly to admission, LUS performed in our patients at discharge was characterized by normal A pattern, mostly represented in the anterosuperior regions, and B pattern in more than one fourth of the acquired videos, mostly localized in the lateral and posterior zones, with higher prevalence of B2 pattern over B1. At discharge our patients had the greatest prevalence of C pattern, which was present in 61.3% of the total 336 collected videos (206/336), regardless of the clinical outcome (death or improvement).

It is noticeable that a difference in LUSS at admission and discharge was not detected, even in survived patients, suggesting that variation of LUSS, which has been validated by Soummer et al. [7] for patients affected by other conditions originating severe hARF, could not be a useful tool in COVID-19 patients. Other recent studies6 seem to demonstrate that the LUSS severity correlates with CT scan and the clinical picture. However, how chest imaging may be related to the evolution of the disease remains still not proven and the trend observed in our patients seems to exclude a role for the traditional LUSS in COVID-19. Indeed, many evidences suggest that the clinical characteristics of COVID-19 related ARDS are different from classical ARDS observed before this pandemic [16]. This could be the reason of our findings: in ARDS caused by other conditions than COVID-19, aeration loss predominates in dependent lung regions (focal ARDS), with parts of upper lobes remaining more aerated [18]. In COVID-19 patients, the presence of non-translobar, mantellary, widespread consolidations leads to an increase in median LUSS and therefore makes the already described LUSS thresholds hardly applicable to guide clinical decisions [7]. Some authors [19] suggest a modified method to calculate LUSS, in order to overcome some limitations of the traditional LUSS and to better quantify the observed findings. Anyway, we believe that the use of the different scoring systems could afflict the absolute value of LUSS but not the difference between the two LUSS values found at admission and discharge.

In the videos collected both at admittance and discharge, lung sliding was always present. In agreement with other authors [12], pleural effusions were uncommon and detected only in one patient who also showed translobar consolidations. Their presence could be due to heart failure or concomitant bacterial pneumonia.

Our study has some limitations. First, the limited sample size does not allow generalization of our results but can only be useful to fix a hypothesis that needs to be confirmed in a large multicentric population study. Moreover, our examination was not optimal as the LUS videos of the posterior paravertebral regions were partially hampered by the supine decubitus in our sedated and mechanically ventilated patients. We believe that in ICU setting, the optimal examination of COVID-19 patients should always include the whole paravertebral areas from the superior to the inferior chest area. Of course, this requires the collaboration of another member of the staff who will be of help in maintaining the patient in lateral decubitus during the posterior examination.

## Conclusions

LUS collected in COVID-19 patients with hARF at ICU admission and discharge appears to be characterized by predominantly, though not exclusively, lateral and posterior non-translobar C pattern and B2 pattern. The calculated LUSS appears to remain elevated at discharge without significant difference from admission in both groups of survived and non-survived patients, suggesting that in COVID-19 patients LUSS could not be as reliable as in non-COVID-19 ARDS patients.

## Data Availability

The datasets analyzed during the current study are available from the corresponding author on reasonable request.
